# Non-immune microenvironmental cues driving melanoma local invasion, migration, and invasive phenotype switching

**DOI:** 10.1007/s10555-026-10398-z

**Published:** 2026-11-12

**Authors:** Gemma Nomdedeu-Sancho, David W. Mullins, Anthony Atala, Shay Soker

**Affiliations:** 1 Wake Forest Institute for Regenerative Medicine, Wake Forest University School of Medicine, Winston-Salem, NC 27101 USA; 2 Department of Microbiology and Immunology, and MD Program, Wake Forest University School of Medicine, Winston-Salem, NC 27101 USA

**Keywords:** Melanoma, Tumor microenvironment, Stroma, Invasion, Phenotype switch, Clinical trials

## Abstract

Melanoma remains the deadliest form of skin cancer and continues to account for a disproportionate number of deaths worldwide. Although targeted therapies have markedly improved outcomes for subsets of patients, both *de novo* and acquired resistance remain major clinical challenges, particularly in tumors with restricted systemic therapeutic options. Central to melanoma progression, invasion, and metastasis is the tumor microenvironment (TME), which engages in dynamic and reciprocal crosstalk with melanoma cells across all disease stages. Here, we synthesize recent advances elucidating how non-immune components of the melanoma TME (including keratinocytes, fibroblasts, extracellular matrix, and adipocytes) mechanistically regulate tumor motility and local invasion. We highlight how biochemical, biomechanical, and metabolic cues cooperatively remodel melanoma invasive behavior. Finally, we review current clinical efforts targeting melanoma-TME interactions and discuss critical gaps that must be addressed to advance these strategies into durable clinical therapies.

## Introduction

Melanoma is the most aggressive type of skin cancer, responsible for the majority of skin cancer–related deaths [1]. It arises from the malignant transformation of neural crest–derived melanocytes located within the basal epidermal layer. Although early-stage *in situ* melanomas are frequently curable by surgical excision, the strong metastatic potential of melanoma underlies most disease-related mortality. Approximately 4% of patients present with metastatic disease at diagnosis, resulting in a marked reduction in 5-year survival from over 99% in localized melanomas to approximately 35% in metastatic disease [1]. However, despite initial surgical control, up to one-third of patients with localized excised melanomas will develop recurrent or metastatic disease [2].

While metastatic melanoma represents the greatest clinical challenge, melanoma *in situ* and early-stage lesions are increasingly recognized as biologically active disease states that can harbor molecular and microenvironmental features predictive of invasion and progression. Understanding early microenvironmental cues that support melanoma persistence and invasion is therefore essential.

Current systemic melanoma treatments include targeted therapies (signal transduction inhibitors) or immunotherapies (immune checkpoint inhibitors [ICI]) [3]. Approximately 50% of melanomas harbor activating BRAF^V600E^ mutations, leading to MAPK pathway hyperactivation [4] and sensitivity to BRAF and MEK inhibitors. However, therapeutic resistance frequently arises, limiting the long-term benefit [5]. Non-BRAF-mutated melanomas, including NRAS-mutated, NF1-mutated, and triple wild-type melanomas, are primarily treated with ICI [3], yet not all patients are suited to receive immunotherapy or can tolerate the related toxicities, and many exhibit primary or acquired resistance [6]. These limitations highlight an urgent need for alternative treatment strategies.

The melanoma tumor microenvironment (TME) includes all the cellular and non-cellular components adjacent to the cancer cells, such as signaling molecules, skin cells, and extracellular matrix (ECM) that dynamically interact with tumor cells to regulate growth, invasion, and therapy response [7, 8]. In turn, the melanoma cells actively remodel their surrounding microenvironment to enhance survival, motility, and dissemination [9–12]. These reciprocal interactions occur sequentially during melanoma progression, with distinct TME components regulating specific phases of tumor progression. Following the radial growth phase (RGP), the initial spreading within the epidermis, melanoma cells breach the collagen IV-based dermal–epidermal junction (DEJ) [13] and invade the underlying dermis, where they interact with fibroblasts [14], remodel the ECM architecture [15], and acquire a migratory phenotype [8, 16, 17]. Deeply invasive melanoma lesions can reach and reprogram hypodermal adipocytes to confer tumor cells with a metabolic advantage [18]. Throughout this process, paracrine signaling, cell–cell interactions, and biochemical cues guide tumor progression and metastatic spread.

The TME also influences phenotype switching, a reversible transcriptional adaptation to intrinsic cues and microenvironmental stressors such as hypoxia [19], inflammation [20], metabolic stress [21], and ECM-mediated physical confinement [22], that provides melanoma with intratumor heterogeneity to support tumor survival, invasion, and resistance to therapies [8, 16, 17, 23, 24]. The transcription factor MITF, the master regulator of the pigmentation pathway in melanocytes, acts as a rheostat of this process and can be used to distinguish the different melanoma states [16]. As MITF levels drop, melanoma cells transition from a differentiated, therapy-sensitive “proliferative” profile (MITF^high^/AXL^low^) to a resistant, undifferentiated “invasive” state (MITF^low^/AXL^high^), while a complete loss of MITF triggers cellular senescence [25]. Melanocytes are not epithelial cells and do not undergo the classical epithelial-mesenchymal transition (EMT) to trigger invasiveness. However, upregulation of EMT-related genes, such as TWIST1 and ZEB1, drives the phenotypic switch and acquisition of the MITF^low^/AXL^high^ invasive state [26]. In addition, dedifferentiated MITF^low^/AXL^high^ cells drive targeted therapy resistance by upregulating growth factor receptors such as AXL, ROR2, and NGFR, which bypass MAPK signaling to maintain cell growth [27]. They also foster an immunosuppressive environment by downregulating melanocytic antigens, secreting proinflammatory cytokines to recruit myeloid-derived suppressor cells (MDSCs) [28], and overexpressing PD-L1, which impairs immunotherapy response [28]. Ultimately, TME-driven fluctuations in MITF allow melanoma to dynamically adapt to treatment pressure and fuel disease progression.

Thus, the TME’s multiple roles in promoting melanoma tumor growth, spreading, and drug resistance make it a novel and interesting therapeutic target. Importantly, these microenvironmental components do not function in isolation, but rather as part of a coordinated and dynamic network. Emerging evidence indicates that stromal compartments converge on shared signaling pathways (including Notch, Wnt, and integrin/FAK signaling), while integrating biochemical, biomechanical, and metabolic cues that collectively regulate melanoma plasticity and invasion. These interactions are highly context-dependent, with spatial and temporal factors shaping whether specific pathways promote or restrain tumor progression.

This review focuses on microenvironmental cues that support melanoma invasion, migration, and phenotype switching, excluding immune-mediated interactions, which have been extensively reviewed elsewhere [29]. While intrinsic oncogenic drivers remain critical determinants of melanoma behavior [4, 30], we emphasize microenvironment-derived signals that enhance cell motility, phenotypic plasticity, and invasion. We also evaluate recent clinical trials targeting melanoma-TME interactions and highlight key gaps that must be addressed to improve translational success.

## Interactions with epidermal keratinocytes: from horizontal spreading to vertical dermal invasion

Keratinocytes constitute the predominant cell population of the epidermis and play a central role in regulating melanocyte homeostasis [31]. Melanocytes and keratinocytes physically interact by forming the epidermal-melanin unit, in which each melanocyte distributes UV-protecting melanin to 36 surrounding keratinocytes via dendritic extensions [32, 33]. These contacts rely on cadherin-mediated adhesion, Notch signaling, and reciprocal paracrine communication [34]. In melanoma transformation, this tightly regulated relationship breaks down: keratinocytes progressively lose control over transformed melanocytes, while melanoma cells actively reprogram adjacent keratinocytes to adopt tumor-supportive functions, facilitating invasion and progression.

### Role of keratinocytes in melanoma initiation

Classically, melanomagenesis has been attributed to the acquisition of driver mutations in proto-oncogenes such as BRAF and NRAS, which hyperactivate the MAPK signaling and promote melanocyte proliferation. However, not all melanocytic lesions harboring driver mutations progress to melanoma [35]. For example, over 80% of benign nevi have BRAF^V600E^ mutations [36] yet remain growth-arrested through oncogene-induced senescence (OIS). Escape from OIS arrest requires additional events, including secondary mutations (for example, PTEN loss), epigenetic reprogramming, and interactions with the surrounding microenvironment [37]. Recent studies have therefore focused on how keratinocytes contribute to senescence escape, identifying microenvironmentally driven mechanisms that may be therapeutically targetable at the earliest states of tumorigenesis.

Tagore and colleagues [38] presented that GABAergic signaling between keratinocytes and melanoma cells may be a critical driver for melanoma initiation, particularly in BRAF^V600E^ contexts. Using zebrafish, mouse, and human skin models, the authors showed that melanoma cells increased GABA production by upregulating GAD1. Melanoma-derived GABA activated GABA-A receptors on keratinocytes, suppressing electrical activity and inducing LIF secretion, thereby promoting unregulated tumor proliferation. Genetic and pharmacologic disruption of this axis markedly impaired melanoma progression, highlighting GABA signaling as a potential early-intervention target. Still, this mechanism fails to fully explain tumor initiation across all contexts, given its absence in BRAF WT melanomas.

A single recent study [39] found that roughly 60% of primary melanomas harbor genomic desmosome mutations that selectively reduce desmosome expression in adjacent keratinocytes but not melanoma cells, as shown by spatial transcriptomics on human tumor samples. Conditioned media experiments revealed that desmosome-deficient keratinocytes can promote melanocyte proliferation through as-yet-unidentified paracrine factors. Complementarily, disruption of keratinocyte polarity through epidermal Par3 loss increased P-cadherin expression at the keratinocyte-keratinocyte junction, favoring homotypic interactions over heterotypic contacts with melanocytes. This shift enhanced ERK/Akt signaling in melanocytes, driving proliferation, dedifferentiation, and metastatic competence. Consistent with these findings, low epidermal PAR3 and high P-cadherin expression correlate with poorer survival in melanoma patients [40].

While the acquisition of additional intrinsic mutations remains the main established mechanism of melanoma initiation, these novel studies collectively underscore the importance of the keratinocyte niche in this process. However, it remains unclear whether melanoma cells actively induce a pro-tumorigenic epidermal environment, or whether pre-existing keratinocyte mutations increase susceptibility to malignant transformation. Resolving this temporal relationship will be critical for defining preventative and early intervention strategies.

### Role of keratinocytes in melanoma spreading in the epidermis

In addition to regulating melanocyte proliferation, keratinocytes restrict melanocyte motility and limit colonization of the basal and suprabasal epidermal layers. Loss of keratinocyte control over transformed melanocytes promotes pro-invasive EMT-like programs in melanoma cells and subsequently facilitates the disruption of the DEJ and dermal invasion. Downregulation of E-cadherin releases melanoma cells from keratinocyte adhesion, favoring interactions with N-cadherin-expressing stromal cells such as dermal fibroblasts [41]. However, neither reduced E-cadherin nor elevated N-cadherin alone reliably predicts patient outcome [42], suggesting that additional keratinocyte-dependent mechanisms regulate early melanoma invasion.

**Fig. 1:**
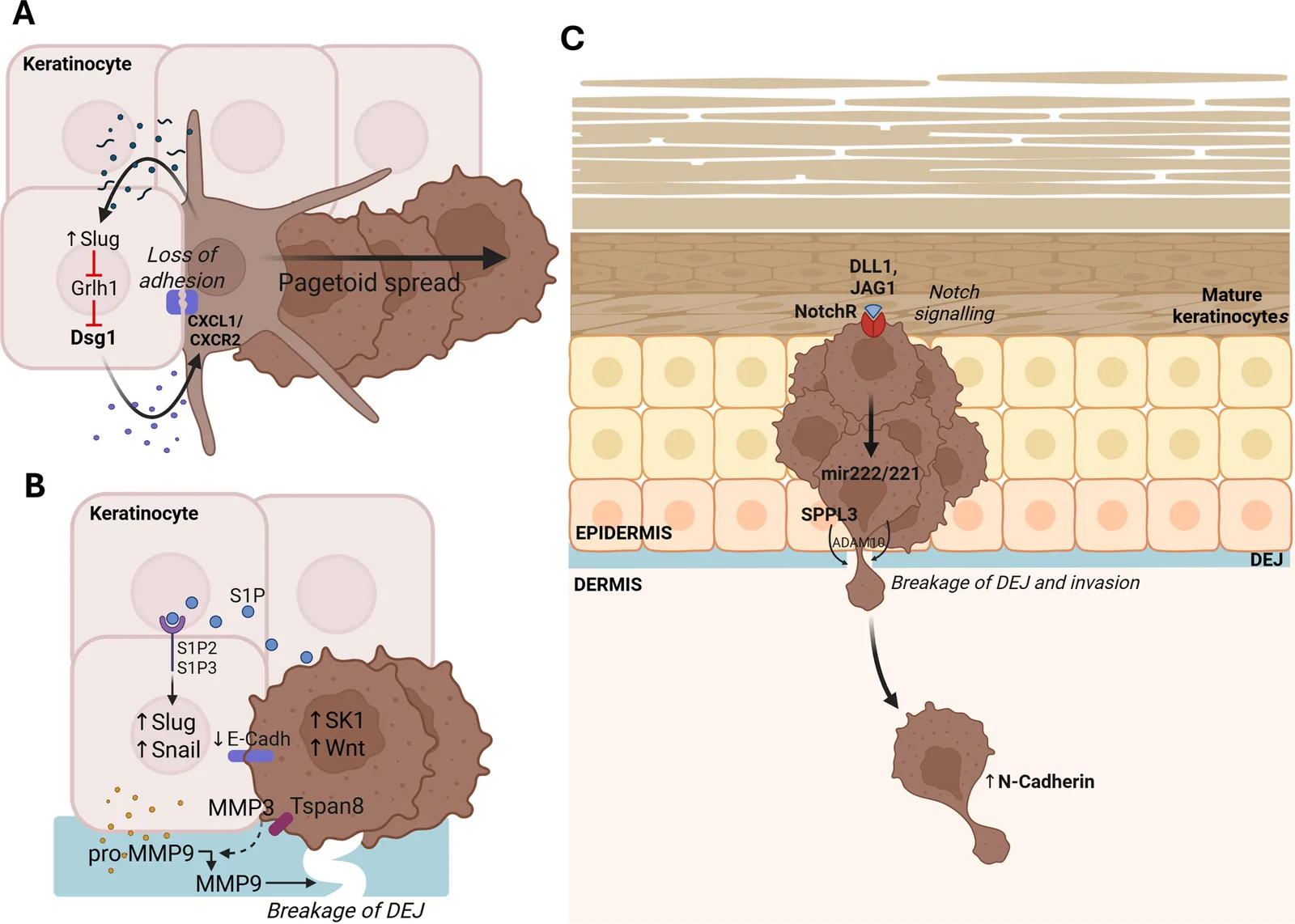
Melanoma-Keratinocyte interactions driving tumor epidermal spreading and dermal invasion. **A** Loss of Dsg1 expression in keratinocytes promotes the radial expansion of melanoma cells within the epidermis through CXCL1/CXCR2 signaling. **B** Paracrine crosstalk between keratinocytes and melanoma cells promotes the breakage of the collagen IV-based dermal-epithelial junction (DEJ) to allow melanoma dermal invasion. Melanoma-derived S1P induces EMT programs in keratinocytes reducing E-cadherin-based adhesion and increasing pro-MMP9 secretion. Tspan8 in melanoma cells may induce MMP3 secretion in keratinocytes, to turn pro-MMP9 into active MMP9, promoting DEJ degradation. Whether physical Tspan8-mediated cell-cell interactions are needed to trigger MMP3 secretion remains unknown. **C** Notch-mediated contacts between melanoma cells and mature keratinocytes trigger dermal invasion and DEJ crossing. In BRAF-mutant melanomas, SPPL3 transfer promotes ADAM10 activation, which may further activate Notch in melanoma cells and break the ECM when secreted to the extracellular space. N-Cadherin-based contacts sustain the interaction between melanoma cells and dermal fibroblasts. Created with Biorender.com

Importantly, melanoma escapes from the spatial control exerted by keratinocytes before the overt loss of E-cadherin expression. Burks and colleagues [43] identified loss of keratinocyte desmoglein-1 (Dsg1) as an early event in melanoma progression that permits intra-epidermal tumor cell movement and primes local invasion within the primary lesion. Using conditioned media, organotypic 3D cultures, and transcriptomics, the authors showed that melanoma cells secrete paracrine signals [44] that upregulate the transcriptional repressor Slug, which inactivates the Dsg1 transcriptional activator Grlh1, reducing keratinocyte Dsg1 levels. In turn, Dsg1-deficient keratinocytes secrete CXCL1, which promotes pagetoid melanoma spread via CXCR2 signaling (Fig. 1A). These findings suggest that melanoma progression can initiate in the epidermis before dermal invasion, revealing a possible therapeutic window. However, the specific melanoma-derived factors that trigger this keratinocyte response remain unidentified. Elucidating those components could provide insights into early progression biomarkers for detecting primary lesions likely to become invasive.

### Role of keratinocytes in melanoma invasion and penetration to the dermis

Loss of Dsg1 alone is insufficient to enable melanoma cells to cross the DEJ, indicating that additional factors govern dermal invasion. Similarly, some melanoma cell lines are intrinsically more invasive [45], and clinically non-aggressive melanomas can metastasize if undiagnosed. This underscores that genetic background alone does not entirely explain invasion and highlights the role of the TME in this process. Co-culture of low aggressive melanoma cells with epidermal keratinocytes induced mesenchymal traits, upregulation of EMT-related genes, and enhanced invasiveness. These effects were associated with activation of Wnt signaling via Nodal [46], a pathway linked to melanoma plasticity and aggressiveness [47]. However, successful dermal invasion ultimately requires physical disruption of the collagen-rich DEJ through collagen-degrading enzymes.

Using reconstructed skin models, Van Kilsdonk and colleagues [48] demonstrated that keratinocytes are a minimal requirement for melanoma cells to breach the DEJ and invade the underlying dermis. When melanoma cells were seeded onto decellularized dermal (DED) matrices, invasion did not occur. In contrast, co-seeding of melanoma cells with keratinocytes enabled dermal invasion, establishing keratinocyte involvement as a requisite for this transition.

Keratinocytes cultured alone on DED matrices secreted pro-MMP9. MMP9 is critical for the degradation of the collagen IV-rich DEJ; however, in this scenario, keratinocyte-derived pro-MMP9 was never converted to its active form. Moreover, melanoma cells exposed to keratinocyte-conditioned media failed to invade, indicating that direct cell–cell contact between melanoma cells and keratinocytes is required to initiate proteolysis-dependent dermal invasion.

A potential contact-dependent mechanism was suggested by El Kharbili and colleagues [49], who identified Tetraspanin-8 (Tspan8) as a key mediator of melanoma dermal invasiveness. Absent in healthy skin but upregulated in aggressive primary melanomas and lymph node metastases [50], Tspan8 belongs to a family of membrane-bound proteins involved in adhesion, signal transduction, migration, and invasion [51]. Tspan8 upregulation was detected after the invasive phenotype switch, decreasing cell stiffness [52] and integrin-b1-mediated matrix anchorage [53]. Beyond enabling cell motility, this reduced adhesion may allow Tspan8 to serve as an independent point of contact with neighboring keratinocytes. In skin equivalent models, melanoma-expressed Tspan8 induced keratinocyte secretion of MMP3, which in turn activated keratinocyte-derived pro-MMP9 at sites of contact. MMP9-induced DEJ degradation was further augmented by reduced TIMP1 secretion by melanoma cells, thereby enhancing MMP9-mediated collagen breakdown and facilitating dermal entry. Although the upstream signals driving Tspan8 and MMP3 upregulation remain unclear, blockade of Tspan8 effectively abrogated invasion, suggesting a therapeutically tractable alternative to direct MMP inhibition, which has proven ineffective clinically [54, 55]. Additional melanoma-keratinocyte interactions converge on MMP9 induction, highlighting molecular redundancy but reinforcing the central role of MMP9 in DEJ degradation. Melanoma overexpression of SK1 increases paracrine secretion of the sphingolipid S1P, which signals through S1P2 and S1P3 receptors in keratinocytes to induce the EMT genes Slug and Snail through the MAPK-PI3K/Akt pathway (Fig. 1B). This reduces E-cadherin expression, liberates melanocytes from keratinocyte control, and stimulates keratinocyte MMP9 secretion [56], jointly promoting DEJ disruption.

Originally, Van Kilsdonk’s study [48] also showed that dermal invasion requires interaction with mature keratinocytes. When keratinocyte maturation was prevented, melanoma cells did not invade the DED but formed rounded nests atop the DEJ, resembling RGP. Airlifting restored keratinocyte differentiation and rescued invasion, recapitulating the vertical growth phase (VGP) and indicating that terminally differentiated keratinocytes determine invasiveness. Golan et al. [57] made similar observations and investigated the RGP-to-VGP transition. Co-culture with differentiated but not basal keratinocytes increased melanoma invasiveness. Human samples showed that VGP cells had active Notch signaling at the contact sites with upper-layer keratinocytes that expressed DLL1 and Jag1. Notch inhibition with DAPT abrogated this effect. Mechanistically, Notch suppresses MITF function [57], which relieves RBPJK-mediated repression of miR222/221, a microRNA cluster linked to migration [58], and triggers invasion. Notch signaling persists after dermal entry, sustaining pro-invasive behavior independent of keratinocyte contact (Fig. 1C).

Nonetheless, these studies did not address how Notch-activated melanoma cells physically penetrate the DEJ. A recent study [59] showed that BRAF-mutated melanoma cells activate and transfer SPPL3 to keratinocytes, inducing ADAM10 activation in both cell types. Besides being a Notch activator through the cleavage of the intracellular portion of the Notch receptor, ADAM10 is also secreted to the extracellular space in vesicles [60] and can modulate cell adhesion and extracellular matrix breakdown, especially collagen IV, linking Notch signaling to DEJ degradation (Fig. 1C). Future studies examining Notch-dependent MMP secretion could clarify how cell–cell and cell–ECM interactions coordinate early DEJ disruption in both BRAF-mutant and BRAF-WT melanomas.

Overall, early melanoma invasion into the dermis is a multicomponent process shaped by the epidermal microenvironment, where parallel and redundant pathways promote reduced adhesion and a mesenchymal, pro-invasive phenotype. Melanoma cells simultaneously reprogram keratinocytes to increase secretion of collagen-degrading enzymes, facilitating DEJ crossing. As melanoma cells penetrate the dermis, upregulation of N-cadherin (Fig. 1C) enhances interactions with dermal fibroblasts [61], priming subsequent migration within the dermal compartment.

## Interactions with dermal fibroblasts

**Fig. 2:**
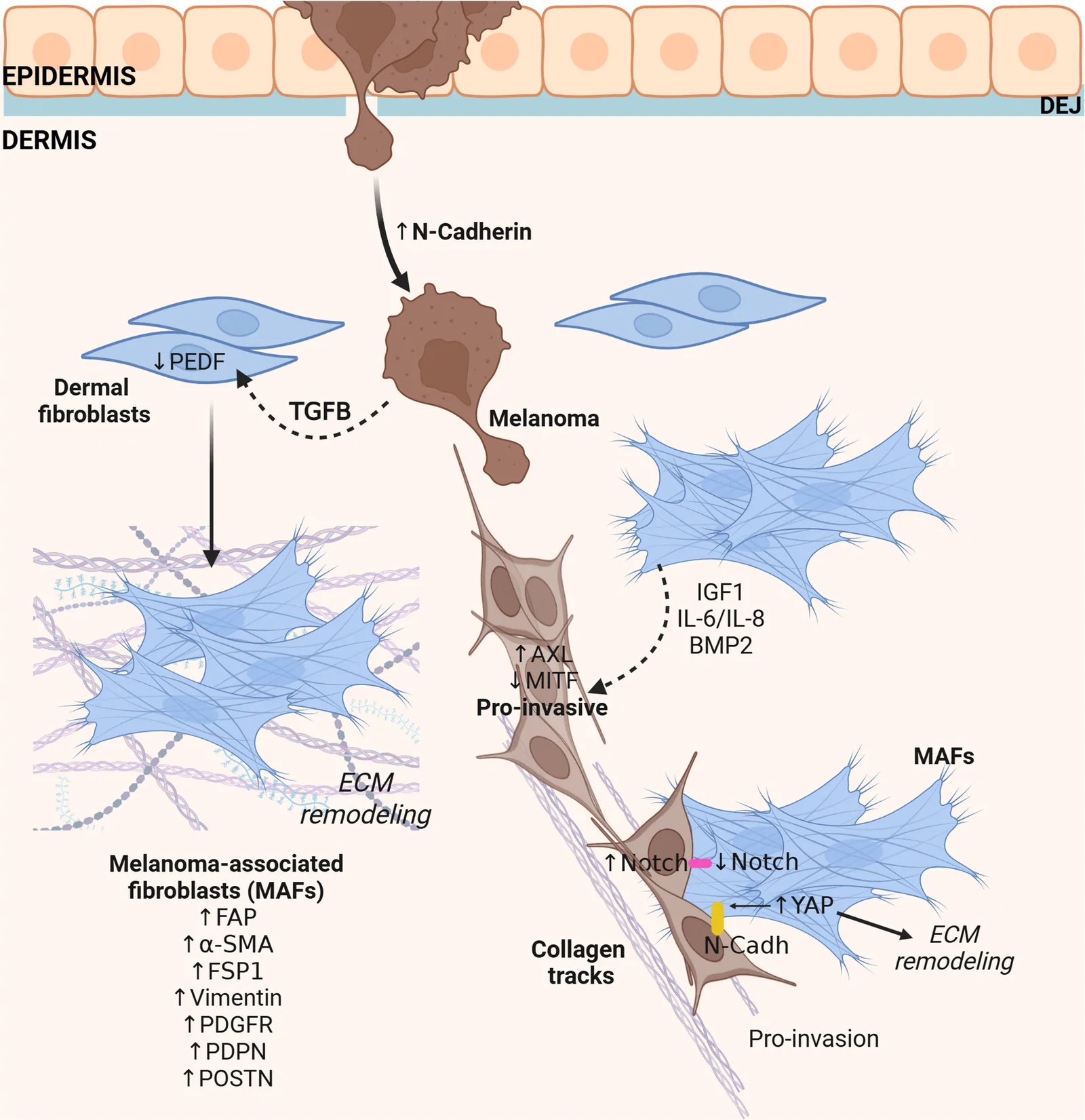
Invasive melanoma cells transform dermal fibroblasts into melanoma-associated fibroblasts (MAFs), which sustain further invasion within the dermis. Signals from melanoma cells, such as TGF-β, induce the acquisition of the MAF phenotype. In early stages of disease, matrix-depositing MAFs (mMAFs) at the tumor border extensively remodel the ECM, promoting melanoma invasion along collagen tracks. In turn, the MAF secretome also exacerbates the MITF^low^/AXL^high^ invasive phenotype of melanoma cells. Cell-cell interactions maintain high pro-invasive Notch signaling in melanoma cells, whereas YAP activation in MAFs induces N-cadherin-based adhesion, enabling melanoma cell mobility. Created with Biorender.com

Melanoma cells in the dermis are stabilized by N-cadherin-mediated interactions with dermal fibroblasts. These interactions promote survival outside their niche via Akt activation, increasing β-catenin, and repressing the pro-apoptotic factor BAD [62]. In physiological skin, normal fibroblasts are quiescent but become activated myofibroblasts after injury, upregulating alpha-smooth muscle actin (α-SMA) [63] increasing contractility and producing ECM to support wound repair. In melanoma, fibroblasts adopt a constitutively activated, apoptosis-resistant state resembling chronic wound healing and become melanoma-associated fibroblasts (MAFs). In addition to α-SMA, MAFs express fibroblast activating protein (FAP), fibroblast-specific protein 1 (FSP-1), platelet-derived growth factor receptor (PDGFR), podoplanin (PDPN), periostin (POSTN), and vimentin [64] (Fig. 2). Melanoma cells induce this fibroblast transition through cell–cell and paracrine interactions [10], while MAFs promote tumor proliferation, invasion, migration, and resistance to therapies [65]. For example, PDPN expression in MAFs correlates with increased tumor thickness, metastasis, and poorer survival [66]. High abundance of MAFs also correlates with drug resistance. Notably, BRAFi treatment induces POSTN secretion by MAFs, via β-catenin; in melanoma cells, POSTN activates the PI3K/Akt signaling, relieving BRAFi-induced ERK inhibition and promoting resistance to therapy [67].

In most tumors, cancer-associated fibroblast (CAF) populations are heterogeneous and dynamic throughout tumor progression, exerting both supportive and suppressive effects [68]. A recent single-cell analysis [69] revealed three main CAF (MAF) subtypes in melanoma: RGS5+ perivascular MAFs, matrix-producing MAFs (mMAFs), and immunomodulatory MAFs (iMAFs). RGS5+ cells are broadly distributed, though their role in tumor progression remains uncharacterized. mMAFs align at the tumor-stroma border and secrete matrix, forming a dense ECM barrier that can restrain initial tumor spreading [70] but also drive therapy resistance by physically blocking drug and immune cell penetration [71, 72]. iMAFs are patchily distributed in the tumor and secrete immune-recruiting and immunosuppressive cytokines. Enriched in aggressive, late-stage tumors, iMAFs may drive T-cell exhaustion, while also providing paracrine signaling to aid melanoma cell invasion (discussed below) [73]. Both MAF populations act synergistically: mMAFs establish a physical barrier around the tumor that excludes therapeutic agents and T cells, while iMAFs induce immune dysfunction, jointly driving disease progression and treatment resistance.

Because these MAF subtypes were not clearly phenotypically defined until recently, this section reviews MAFs’ general effects on tumor progression. Although iMAFs predominantly secrete cytokines and chemokines, and mMAFs drive melanoma-MAF interactions at the tumor margin, future research needs to map the following signaling factors and ligand-receptor interactions to specific MAF subsets.

### Transformation of dermal fibroblasts into melanoma-associated fibroblasts (MAFs)

Continued melanoma signaling drives normal fibroblasts toward a tumor-promoting phenotype. Co-culture studies showed that melanoma cells stimulate MAF activation and proliferation via TGFa-EGFR signaling, while contact-dependent mechanisms further enhance invasion [74]. Additional melanoma-derived factors, including FGF, interleukins, and microvesicles, also contribute to this process [75]. Importantly, BRAFi such as vemurafenib can activate fibroblast MAPK signaling off-target, promoting MAF conversion and secretion of HGF [76], which in turn supports the E-to-N-cadherin switch and Dsg1 dysregulation [62], reinforcing invasion. These findings highlight the need to consider TME-mediated effects when designing therapies.

### Cell–cell interactions driving melanoma dermal migration

During VGP, melanoma cells preferentially interact with dermal fibroblasts via N-cadherin. MAFs play a central role in maintaining these interactions and promote tumor growth and migration. YAP1 expression in MAFs regulates N-cadherin-mediated adhesion and tumor dynamics. In BRAF-mutant models, YAP1 depletion in MAFs reduces melanoma growth and N-cadherin expression in both stromal and tumor cells, whereas YAP1 activation enhances tumor progression [77]. Reduction of tumoral N-cadherin following YAP1 depletion in MAFs disrupts cell–cell adhesion and signaling, thereby limiting melanoma survival and proliferation. Concurrently, YAP1 loss also impairs ECM remodeling, reducing invasion. Although the mechanisms linking MAF YAP1 to melanoma N-cadherin remain unclear, targeting stromal N-cadherin interactions may reverse EMT and inhibit dermal invasion.

### The role of MAFs’ secretome on melanoma dermal migration

MAF-derived secreted factors also play a crucial role in melanoma progression. Activated MAFs secrete IGF1, promoting early melanoma proliferation, survival, and migration via MAPK and Akt signaling [78]. MAPK activation also results in increased expression of tumoral IL-8 [62], an interleukin whose expression has been shown to correlate with poorer overall survival [79]. However, at later stages, melanoma cells become unresponsive to MAF-secreted IGF1.

MAFs produce pro-inflammatory cytokines that enhance invasion. In 3D spheroid models, MAF-derived IL-6 and IL-8 increased melanoma migration and invasion without affecting proliferation. Dual blockade of these cytokines abrogated the effect [73]. Interestingly, sensitivity depended on tumor genotype, with BRAF-mutant cells being particularly responsive to this treatment, suggesting crosstalk between MAPK and cytokine signaling. Based on these findings, combining inhibition with targeted therapies may enhance their efficacy and help prevent resistance.

Finally, systemic physiological features of fibroblasts also affect tumor progression. Chhabra et al. [80] revealed that sex-specific changes in skin fibroblasts also dictate melanoma invasion and drug tolerance. Aged male fibroblasts exhibited increased oxidative stress and released higher levels of BMP2, driving the slow-cycling AXL^high^ invasive phenotype associated with drug resistance [16, 17, 80]. BMP2 inhibition reversed this phenotype and sensitized melanoma cells to BRAFi and MEKi [80], underscoring the need for sex- and age-tailored research in melanoma. Additionally, MAFs remodel the ECM through metalloproteases and proteins such as CCN2 and POSTN that activate MMPs, further facilitating invasion [81] (Fig. 2). ECM remodeling also greatly affects melanoma progression and invasion. Cell-ECM interactions driving this process are discussed in the following section. 

## Interactions with hypodermal adipocytes

**Fig. 3:**
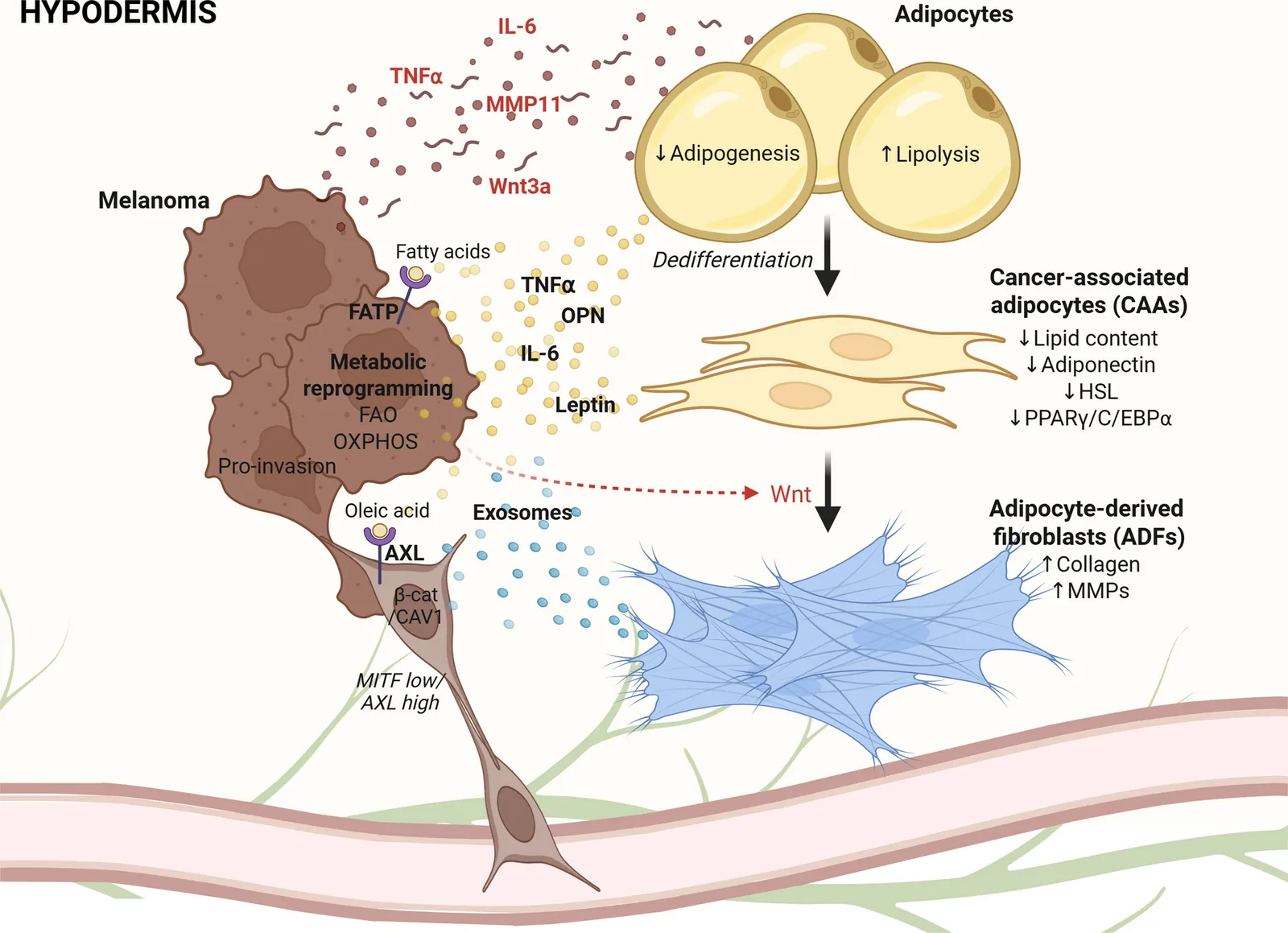
Melanoma cells that reach the hypodermis transform adipocytes into cancer-associated adipocytes (CAAs) and adipocyte-derived fibroblasts (ADFs), which then support further invasion towards blood vessels and lymphatics. Studies in other cancers reveal that the secretion of factors, shown in red, reduces adipogenesis and enhances lipolysis in adjacent adipocytes, thereby driving their dedifferentiation into CAAs. Further contacts between tumor cells and CAAs can lead to the production of ADFs. These findings have yet to be confirmed in melanoma. CAAs secrete adipokines, metabolites, and extracellular vesicles that metabolically reprogram melanoma cells, support their growth, and drive their invasion. Created with Biorender.com

As melanoma invades vertically, tumor cells reach the subcutaneous adipose tissue, the hypodermis (Clark level V), which consists primarily of triglyceride-storing adipocytes that release fatty acids and molecules into the microenvironment [11]. These include adipokines such as IL-6, IL-8, and CCL2, metabolic markers such as IGF-1, and hormones such as leptin and adiponectin [11]. Melanoma cells express receptors for these factors [82], and upon binding, they activate survival, proliferation, and migration pathways, including PI3K/Akt, MAPK, and JAK/STAT [83]. Clinically, increased adiposity correlates with faster progression, reduced treatment response, and higher mortality [84]. Like fibroblasts, adipocytes can be reprogrammed into cancer-associated adipocytes (CAAs). This process involves dedifferentiation and the acquisition of a tumor-supportive phenotype [85], characterized by the loss of differentiation markers such as hormone-sensitive lipase (HSL), adiponectin, and PPARγ [86], and a reduction in lipid content associated with increased lipolysis. At the same time, CAAs upregulate pro-inflammatory cytokines such as TNFα and IL-1β and release MMPs, which further exacerbate ECM remodeling [86]. Ultimately, akin to the mechanism described for breast cancer, persistent tumor-adipocyte interaction could further drive their conversion into adipocyte-derived fibroblasts (ADFs) via Wnt3a signaling, yielding cells with a CAF-like phenotype and a pro-invasive secretome [87]. Nonetheless, this remains to be verified in melanoma (Fig. 3).

### Transformation of normal hypodermal adipocytes into cancer-associated adipocytes (CAAs) and adipocyte-derived fibroblasts (ADFs)

Co-culture studies showed that melanoma cells induce adipocyte dedifferentiation within days, producing CAAs and ADFs with reduced adipocyte-specific gene expression and increased collagen and MMP production [11, 85]. The metabolic reprogramming of adipocytes by melanoma cells, which turns them into tumor-supportive cells, suggests that CAAs are potential therapeutic targets for melanoma. While the specific mechanisms by which melanoma cells reprogram hypodermal adipocytes remain incompletely defined, studies in other cancers, mainly ovarian and breast cancer, provide insights. For example, tumor-derived TNF-a suppresses PPARγ, increasing lipolysis and promoting ADF formation [88], while IL-6 enhances lipolysis *in vivo* and *in vitro*, therefore enhancing fat mobilization and catabolism [89]. TME-induced activation of MMP11 negatively regulates adipogenesis [90]. In breast cancer, Wnt3a activates β-catenin, upregulates Wnt10b, and inhibits adipogenic transcription factors (PPARγ, C/EBPα), driving dedifferentiation and ADF formation [87]. Similar effects were observed with Wnt5a in pancreatic cancers [91]. Inhibition of Wnt can reverse this phenotype and restore lipid accumulation, suggesting the possibility of using Wnt inhibitors as a therapeutic approach [92]. Tumor-derived exosomes also contribute by suppressing adipocyte markers and promoting lipolysis.

While none of these mechanisms has been confirmed in melanoma, effectors such as IL-6, TNF-a, and Wnts are known to be expressed by melanoma cells. Therefore, similar mechanisms may also explain the melanoma-induced transformation of hypodermal adipocytes into CAAs. Validating these findings in melanoma would help identify targets that disrupt adipocyte-driven tumor progression.

### Role of CAA’s secretome on melanoma migration

Unlike other stromal interactions, melanoma migration does not require direct adipocyte contact [93]. Instead, melanoma cells exploit adipocyte-derived factors to reprogram their metabolism and enhance their capacity to spread.

Adipokines are the main factors released by adipocytes and CAAs. Adipocyte-secreted leptin stimulates melanoma growth, likely via MAPK and Akt activation [94]. While leptin has been linked to chemotherapy resistance in other cancers [95], this is yet to be proven for melanoma. Leptin may also promote migration, as elevated serum leptin correlates with metastases in the sentinel lymph node [96]. CAAs and ADFs also secrete OPN, which enhances invasion, stimulating integrin signaling and MMP2 activation. Adiponectin may have contradicting effects in different tumors [97, 98]. Low adiponectin was linked to increased uveal melanoma aggressiveness [99]; however, it can also suppress MITF [100]. Whether this inhibition favors the acquisition of a pro-invasive MITF^low^/AXL^high^ melanoma phenotype warrants investigation.

Adipocyte-derived cytokines can also promote invasion in early disease, even before penetration into the hypodermis. In RGP lesions, adipocytes infiltrate the dermis and secrete IL-6 and TNF-α, suppressing miR-211 in melanoma cells and increasing TGF-β receptor expression. This sensitizes cells to TGF-β and induces a phenotypic switch toward the invasive state[101]. This effect was reversed by TGF-β inhibition, suggesting a promising strategy to intercept melanoma local invasion at its earliest stages.

Although cancer cells are classically glycolytic, adipocyte-derived fatty acids can reprogram melanoma cells toward lipid utilization. Fatty acids are taken up via fatty acid translocase protein (FATP) and used for energy production and membrane remodeling, supporting invadopodia formation and migration [102]. FATP receptor overexpression is associated with poor prognosis in breast and ovarian cancers [103, 104]. For melanoma, *in vitro* co-cultures with adipocytes increased melanoma cell line proliferation, invasion, and ECM degradation via FATP-mediated fatty acid transfer [105]. *In vivo* FATP1 overexpression enhanced invasion, whereas inhibition reduced lipid uptake and invasive capacity [102], highlighting FATP's potential as a therapeutic target. However, FATP-independent pathways also exist, which may render FATP inhibitors ineffective. In MITF^low^/AXL^high^ melanoma, adipocyte-derived oleic acid promotes invasion independently of FATP [18]. These cells induce Wnt5a-driven lipolysis in CAAs and take up the released fatty acids via the AXL receptor. Signaling through AXL recruits SRC and promotes the nuclear localization of the β-catenin-CAV1 complex, driving EMT-related gene expression and exacerbating the invasive phenotype [18]. This pathway highlights the mechanistic importance of AXL in invasive, therapy-resistant melanoma beyond the transcriptomic state. Although the role of the AXL/SRC/CAV1 axis in melanoma invasion was experimentally demonstrated using omental adipocytes, evidence of increased nuclear CAV1 in melanoma cells close to adipocytes in human samples supported its relevance to melanoma that spreads to the hypodermis, suggesting its use as a therapeutic target [18]. Fatty acid oxidation (FAO) further enhances invasion by inhibiting autophagy and promoting β1-integrin recycling, facilitating focal adhesion turnover and motility [106] (Fig. 3).

In addition to fatty acids, melanoma cells respond to other CAA-derived lipids that influence metastatic behavior [107]. Young adipocyte secretome, rich in phosphatidylcholines, increased OXPHOS and PI3K signaling, favoring melanoma proliferation while inhibiting its migration. Conversely, the aged adipocyte secretome, rich in ceramides, downregulated OXPHOS and increased IL-6 secretion in melanoma cells, resulting in enhanced liver metastasis. Downregulation of OXPHOS in mice carrying young CAA-exposed melanoma cells increased metastatic spread, indicating that the metastatic potential of these cells was mediated by the tumor's metabolic state [107].

CAAs play a central role in melanoma progression by promoting metabolic rewiring, ECM remodeling, and invasion. However, the link between lipid metabolism and cytoskeletal dynamics remains incompletely defined. Elucidating this crosstalk may enable the development of therapies that disrupt adipocyte-driven melanoma migration.

## Melanoma interactions with the extracellular matrix (ECM)

### Dermal ECM composition and remodeling during melanoma progression

Beyond communication with MAFs, melanoma cells rely on interactions with a dynamically remodeled ECM to migrate toward the vasculature and disseminate. The dermal ECM is composed of collagens (types I, III, VII, XV, XVIII), laminin, tenascin-C, fibronectin, hyaluronan, proteoglycans, and integrin ligands, providing a structurally and biochemically active environment for cells to interact with and modify their adhesion and motility properties [108]. During melanoma progression, ECM remodeling intensifies, altering matrix composition and facilitating invasion. In melanoma *in situ*, basement membrane fragmentation increases collagen IV deposition. As the tumor progresses, further disruption leads to the accumulation of collagen IV and laminin, while collagens I, III, and VI surround invasive nests. In advanced stages, the basement membrane is lost, collagen IV decreases, and the proteoglycan versican accumulates at the tumor base, promoting invasion. These progressive changes are coupled with increased angiogenesis and matrix stiffness due to collagen thickening and fibrosis. Melanoma cells sense these changes through surface receptors and activate signaling pathways that enhance motility, which enhances metastatic potential [108]. Both MAFs and melanoma cells can remodel the surrounding ECM in response to TME-derived cues, including soluble factors (discussed above), hypoxia, and mechanical cues. Notably, invasive MITF^low^/AXL^high^ melanoma cells can also autonomously remodel their ECM, generating aligned, stiff collagen networks that facilitate invasion [109].

### Melanoma ECM sensing and movement

Specialized transmembrane receptors on melanoma cells, including integrins, discoidin domain receptors (DDRs), and syndecans, sense biochemical and mechanical ECM cues, triggering intracellular signaling cascades that regulate cell proliferation, survival, and migration.

Integrins are heterodimeric receptors that mediate bidirectional signaling between the ECM and the cytoskeleton. Inside-out signaling alters integrin conformation in response to intracellular ligands to enhance ECM binding, while outside-in signaling occurs upon ECM ligand engagement, typically via RGD motifs in collagen fibers. Activated integrins recruit talin, kindlin, and focal adhesion proteins such as focal adhesion kinase (FAK), vinculin, and paxillin. The formation of focal adhesions (FAs) and FAK binding to Src activate pathways controlling cytoskeletal dynamics, survival (PI3K, Hippo), and migration (Rho/Rac/Cdc42), driving lamellipodia and filopodia formation [12] as well as MMP secretion to further facilitate ECM degradation and invasion [110].

Altered integrin expression has been associated with melanoma progression [111]. Low Sox10 levels in invasive melanoma cells increase α3 integrin and EphA2 expression, promoting adhesion and motility, respectively [112]. Integrin αVβ3 is upregulated during the switch from RGP to VGP [111], and blocking it, along with integrin β1, reduced melanoma adhesion and migration. Integrins can also cooperate with cadherins to regulate adhesion. Integrin α2β1 binds collagen I and interacts with N-cadherin to activate FAK and promote tumor invasion without affecting proliferation [113]. Moreover, stromal cells can further amplify integrin signaling: MAF-derived OPN contains RGD motifs that “trick” cells and enhance avb3 and avb5 signaling, promoting aggressive phenotypes [114]. Integrins also mediate mechanotransduction by sensing increases in matrix stiffness and inducing EMT-associated gene expression [111].

Given its central role in integrin signaling transduction, FAK has been explored as a therapeutic target. In melanoma, FAK promotes progression and resistance to MAPK-targeted therapies and is upregulated following treatment [115–117]. However, FAK inhibitors have shown limited clinical success [111]. Notably, FAK inhibition can reduce migration but, paradoxically, also increase matrix degradation and invadopodia formation. Targeting FAK-paxillin interactions mitigated both effects, suggesting protein–protein interaction inhibitors may offer improved therapeutic strategies [117].

Discoidin domain receptors (DDR1 and DDR2) are collagen-binding receptor tyrosine kinases that regulate melanoma progression. DDR1 binds collagen type I and IV collagen and is enriched in epithelial cells; DDR2 binds collagens I and III and is associated with mesenchymal cells [118]. Upon ligand binding, DDRs trigger signaling cascades that promote migration and invasion. DDR1 enhances collagen fiber alignment via myosin IIA recruitment [119], and its overexpression is correlated with poor prognosis [120]. Inhibition of DDR1 reduced adhesion, MMP secretion, and migration of melanoma cell lines [121]. DDR1 can also interact with integrins and Notch signaling to regulate invasion and chemoresistance [118]. DDR2 signaling activates ERK and stabilizes EMT-related genes such as Snail1, promoting invasion [122]. It also increases MMP2 and MMP9 secretion, enhancing ECM degradation and migration [123].

Integrin, FAK, and DDR inhibitors are currently under investigation as monotherapies to prevent metastasis or in combination with targeted and immunotherapies to prevent treatment resistance. However, clinical efficacy has been limited, likely due to pathway redundancy and compensatory signaling within the TME. Effective strategies will likely require a combinatorial approach targeting multiple nodes of ECM–tumor interaction.

## Tumor-restraining actions of the melanoma TME

The TME is a highly dynamic niche. Although it generally favors tumor progression, the activation of certain signaling pathways can yield opposite outcomes depending on context, cell type, and timing. In early stages, the normal or mildly transformed stroma restrains tumor growth. Often, the same molecular pathways that enforce this restraint early on can switch to promote invasion and progression under different contexts.

In early disease stages, EMT-like transcriptomic programs in melanoma cells drive invasion; however, they can be tumor-restrictive when expressed in niche cells such as epidermal keratinocytes. Using zebrafish and human co-cultures, Ma and colleagues [124] showed that early transformed melanocytes induced EMT-like changes in adjacent keratinocytes, resembling wound healing. Upregulation of the EMT marker Twist in keratinocytes strengthened jam3b-jam3b adhesion to melanoma cells. Paradoxically, this trapped the tumor cells in the epidermis, reducing invasion and boosting survival without altering tumor initiation. Although how Twist drives this adhesion is unclear, uncovering this mechanism could yield strategies that stabilize keratinocyte–melanoma interactions to prevent early spread.

Similarly, in early dermal invasion, normal fibroblasts can suppress melanoma progression by inhibiting EMT-related gene networks in melanoma cells, inducing cell cycle arrest via the MAPK/ERK and Rb pathways [125] and secreting PEDF, which limits tumor growth, angiogenesis, and spread. However, sustained melanoma–fibroblast interaction subverts this suppression. Exposure to melanoma-derived TGFb sensitizes fibroblasts to PDGF-BB, reducing PEDF via JNK1/2 and p38 signaling pathways. PEDF loss promotes the acquisition of a MAF-like phenotype and enhances melanoma cell motility and metastasis *in vivo* (Fig. 2), suggesting that targeting PDGF-BB/TGF-β signaling or restoring PEDF could prevent MAF transformation [126].

Notch signaling is tumor-promoting in melanoma cells, but it exerts a tumor-suppressive effect when expressed in dermal fibroblasts. Compared to normal fibroblasts, MAFs display reduced Notch activity [127]. *In vivo* re-activation of Notch signaling in MAFs resulted in reduced local invasion and distant metastases, in a process involving fibroblast-secreted WISP1, a downstream effector of Notch [128]. Therefore, restoring Notch signaling in MAFs may represent a therapeutic opportunity. Nonetheless, given Notch’s pro-invasive role in melanoma cells, selective MAF targeting remains challenging.

How Notch signaling is downregulated during fibroblast activation remains unclear. One compelling possibility is lateral inhibition driven by melanoma-fibroblast interactions. While lateral inhibition is a fundamental mechanism for determining cell fate during embryonic development, its role in driving tumor progression remains largely unexplored, despite the deep similarities between developmental and oncogenic programs. Research in glioblastoma models showed that co-culture of glioblastoma cells expressing increased Notch ligand Jag1 with low-ligand cells resulted in lateral inhibition: high-ligand cells suppressed their own Notch signaling, while the low-ligand cells experienced an increase in Notch signaling [129]. While this has not been shown in melanoma, similar Notch-mediated interactions may occur. Invading melanoma cells have sustained Notch signaling following interaction with keratinocytes prior to VGP [58] and may express high levels of Notch receptors. Conversely, normal dermal fibroblasts express Notch ligands [130]. Thus, melanoma–fibroblast interactions could suppress fibroblast Notch signaling, promoting a tumor-permissive phenotype. Receptor blocking experiments could help elucidate this process.

ECM mechanics have also been shown to constrain tumor spreading in some contexts. As increased ECM stiffness, driven by activated MAFs, triggers melanoma invasion via FAK signaling and downstream pro-motility programs, one might expect melanoma invasion to increase linearly with stiffness. However, studies show that cell movement peaks at intermediate stiffness and is hindered by both too soft and too stiff matrices [109]. Finally, signaling through DDRs could also have contact-dependent tumor-suppressive roles, including the induction of cell cycle arrest and E-cadherin expression and the downregulation of pro-migration factors like Cdc42, as demonstrated in experiments in epithelial cells [131]. These findings reveal a TME-driven functional plasticity that warrants further investigation in melanoma, particularly to define optimal therapeutic windows that maximize tumor-restraining signaling while suppressing tumor-promoting pathways.

## Targeting components of the TME to prevent melanoma spreading and drug resistance

Beyond actively supporting invasion and motility, the TME also drives resistance to current standard of care therapies, mainly by secreting factors and cues that promote the acquisition of an MITF^low^/AXL^high^ drug-tolerant phenotype or the reactivation of the MAPK signaling cascade. For example, fibroblast secretion of BMP2 enriches the slow-cycling drug-resistant AXL^high^ phenotype [16, 17], whereas fibroblast-derived POSTN [67] and adipocyte-secreted leptin [94] bypass MAPK inhibition by activating AKT signaling. A remodeled ECM induces increased FAK transduction, which can reactivate MAPK and signal through PI3K to overcome BRAF and MEK inhibition [115–117]. Finally, a stiffer ECM can also compromise drug delivery and immune cell infiltration, rendering targeted and immunotherapies ineffective. Given the role of various components of the melanoma TME in tumor invasion and treatment tolerance, therapies targeting stromal interactions are currently under investigation. Although many agents have shown promise in pre-clinical models, only a few have advanced to clinical trials (Table 1).

**Table1:** Clinical trials exploring TME-directed therapies for melanoma. *mAB* monoclonal antibody

Targets	Clinical Trial ID	Phase	Treatments	Indication	Molecular selection	Outcomes	Completion date	Reference
**Notch pathway inhibitors**	NCT01120275	II	RO4929097 monotherapy (gamma-secretase inhibitor)	Stage IV cutaneous melanoma	No molecular selection	Drug levels insufficient to reach Notch inhibition. Minimal clinical activity against metastatic melanoma	2015-08	Lee et al. (2015)
	NCT01196416	Ib/II	RO4929097 + cisplatin + vinblastine + temozolomide	Recurrent or metastatic melanoma	No molecular selection	No significant increase in overall survival or progress-free survival. Drug combination increased toxicity profile	2015-08	-
**Wnt pathway inhibitors**	NCT04851119	I/II	Tegavivint (NSC#826393) monotherapy	Patients under 30 years old with recurrent or refractory solid tumors, including melanoma	No molecular selection in melanoma	Not completed	2028-06	Whittle et al. (2023)
**FAK inhibitors**	NCT04720417	II	Defactinib (VS-6063) + avutometinib (VS-6766 (CH5126766))	Metastatic uveal melanoma	No molecular selection	Terminated early. Patients did not show a significant reduction in disease	2024-04	Seedor et al. (2024)
	NCT06194929 (DETERMINE)	I/II	Defactinib + avutometinib +/− encorafenib	Cutaneous melanoma with confirmed brain metastases	Cohort A: RAS, BRAF, NF1 mutation or triple wild-type status. Cohort B: BRAF mutations (V600E/K)	Not completed	2030-01	-
**Integrin Inhibitors**	NCT00369395	II	Volociximab (mAB against integrin ɑ5β1)	Stage III and Stage IV unresectable melanoma	No molecular selection	Terminated due to insufficient clinical activity	2008-03	Cranmer et al. (2006)
	NCT00066196	II	MEDI-522 (mAB against integrin ɑvβ3) +/− dacarbazine (DITC)	Unresectable, Stage IV metastatic melanoma	No molecular selection	Integrin inhibition combined with DITC was not more effective than DITC alone	2007-06	Hersey et al. (2010)
**c-KIT Inhibitors** *(could potentially inhibit DDRs)*	NCT00667953	I/II	Imatinib (Gleevec) + temzolomide	Unresectable or metastatic melanoma	No molecular selection	Terminated due to difficulty enrolling patients. Drug synergy was not detected. Imatinib was only effective in a small subset of patients, typically those with acral or mucosal melanoma	2011-07	-
	NCT00470470	II	Imatinib monotherapy	Stage III and Stage IV acral or mucosal melanoma	c-KIT mutation or amplification. Prospective patient stratification	Imatinib was effective for patients with KIT mutations with or without amplifications but not for patients with KIT amplifications only	2014-12	Carvajal et al. (2011)
	NCT00424515	II	Imatinib (Gleevec) monotherapy	Stage IV metastatic melanoma—mucosal or acral origin	Documented c-KIT mutation, amplification or both. Post-hoc patient stratification	Imatinib was effective for patients with KIT-mutations with or without amplifications but not for patients with KIT amplifications only	2011-07	Hodi et al. (2013)
	NCT04598009	II	Imatinib + binimetinib (MEKi)	Unresectable Stage III or Stage IV KIT-mutant melanoma	Documented c-KIT mutation	Not completed	2027-05	Tsai et al., 2025
**MMP Inhibitors**	NCT00001683	I	COL-3 (NSC-683551) monotherapy	Refractory metastatic cancer, including melanoma	No molecular selection in melanoma	Limited clinical efficacy. Side effects included photosensitivity	2003-08	Rudek et al. (2001)
**TGF-B Inhibitors**	NCT00356460/NCT00923169	I	GC1008 (mAb against TGF-B)	Renal cell carcinoma or melanoma	No molecular selection in melanoma	Preliminary evidence of antitumor activity. Development stopped due to skin toxicities	2010-01	Morris et al. (2014)

Drugs that inhibit the Notch pathway continue to be studied for a wide range of tumors [132]. RO4929097, a gamma-secretase/Notch signaling pathway inhibitor, showed limited efficacy in metastatic melanoma patients when used as a monotherapy (6-month progression-free survival rate ~ 9%) and failed in combination with chemotherapies due to toxicities. [133]. Future studies will likely investigate Notch inhibitors in combination with standard of care targeted or immunotherapies.

Targeting of the Wnt pathway remains challenging due to its ubiquity. The use of the Wnt inhibitor Tegavivint, which promotes β-catenin degradation, is under study in pediatric patients with recurrent or refractory tumors, including melanoma. While it appears to be well tolerated, the specific outcomes remain to be determined [134]. If effective, the next step would be to use it in adults as monotherapy and in combination with other systemic treatments.

FAK inhibitors (FAKi) are among the most promising TME-directed drugs in development for melanoma and other cancers. The FDA just approved the use of the FAK inhibitor defactinib in combination with the RAF/MEK clamp inhibitor avutometinib for KRAS-mutated low-grade serous ovarian cancer [135]. Despite promising pre-clinical data [115, 136], Avutometinib plus defactinib showed limited clinical efficacy for metastatic uveal melanoma [137]. Ongoing studies are evaluating the effect of their combination with ENCORAFENIB when treating cutaneous melanoma brain metastases. If ineffective, their combination with FAK proteolysis-targeting chimera (PROTAC) degraders could be used to address resistance to FAKi [138].

Integrin-targeting antibodies, such as volociximab (blocking integrin ɑ5β1) and MEDI-552 (against integrin ɑVβ3), have shown minimal clinical benefit alone [139] and in combination with dacarbazine [140], respectively. Co-targeting integrins and FAK may offer improved efficacy or could be used to overcome FAKi resistance [111].

Imatinib is an FDA-approved KIT inhibitor widely used to treat leukemias and gastrointestinal tumors [141]. Imatinib has shown clinical benefit in KIT-mutant mucosal melanomas but limited efficacy in unselected populations [142–144]. Ongoing trials combining imatinib with MEK inhibitors aim to enhance responses and prevent resistance to targeted therapies [145].

Interestingly, imatinib can also block DDR1, one of the main collagen receptors on the surface of melanoma cells; however, this specific pharmacological activity remains unexplored. Targeting DDR alongside integrin/FAK signaling could be worth investigating as a novel approach to prevent the spread of melanoma.

MMP inhibitors to prevent ECM remodeling have historically failed as monotherapies, despite promising pre-clinical data. This is likely due to a lack of specificity and pathway redundancy [54, 146, 147]. Future strategies should emphasize the development of selective inhibitors and their use in combination therapies with targeted or immunotherapies. The anti-TGF-β monoclonal antibody fresolimumab showed early promise, but its development for melanoma treatment was halted due to severe skin toxicities [148].

Overall, most TME-targeting strategies have shown limited clinical efficacy, largely due to pathway redundancy and compensatory signaling, leading to the termination of most programs by phase II. Current efforts focus on combinatorial approaches that integrate TME-directed agents with targeted therapies and immune checkpoint inhibitors. These strategies aim to simultaneously disrupt tumor-intrinsic and microenvironmental support mechanisms, potentially improving outcomes and overcoming resistance in advanced melanoma.

## Conclusion and future perspectives

Melanoma progression is heavily influenced by the TME, where coordinated signaling drives the transition from epidermal confinement to invasion into the dermis and hypodermis, and then toward blood vessels for systemic dissemination. Melanomagenesis is initiated by oncogenic mutations (e.g., BRAF, NRAS) and reinforced by microenvironmental cues that sustain survival, plasticity, and resistance. The TME helps maintain this tumorigenic phenotype by providing pro-survival signals, metabolic substrates, and transcriptional programs that stabilize invasive states and buffer against therapeutic stress.

Although many melanoma-TME interactions are therapeutically targetable, redundancy in signaling pathways remains a major barrier. However, this could be exploited by targeting key regulatory nodes whose inhibition could disrupt progression at multiple stages. For example, Notch signaling promotes early epidermal escape and sustains dermal invasion, while Wnt signaling drives EMT-like events and later could support adipocyte dedifferentiation, enabling deep invasion. FAK acts as a central integrator of ECM cues and intracellular signaling, linking environmental inputs to migratory behavior in a manner that can also be influenced by tumor genotype.

Despite substantial research advances, relatively few TME-directed therapies have advanced to clinical trials, and the results have been modest, at best, particularly with monotherapies or combinations involving chemotherapy. As melanoma treatment increasingly relies on targeted therapies and immunotherapy, future approaches will require a shift toward integrative regimens that simultaneously target shared regulatory hubs across multiple TME compartments rather than individual pathways in isolation. Incorporating spatial and molecular biomarkers of stromal composition, ECM architecture, and metabolic state into trial design may enable more precise patient stratification and improve therapeutic efficacy.

Clinical translation remains challenging, as combination therapies are often limited by toxicity. Effective development of TME-directed strategies also requires preclinical models that accurately recapitulate tumor–stroma interactions. However, current systems face a “complexity-throughput” trade-off: simpler models lack physiological relevance, whereas complex models are not sufficiently scalable for high-throughput drug testing [149]. Refining these platforms is needed to identify the most promising combinations and streamline the translation process.

Overall, expanding therapeutic focus beyond tumor-intrinsic pathways to include the surrounding microenvironment offers a path to overcoming resistance and treatment failure. TME-targeted strategies may be particularly valuable for patients with BRAF wild-type melanoma or those who are refractory to immunotherapy. By disrupting both intrinsic oncogenic signaling and extrinsic stromal support, these approaches have the potential to enable the next generation of effective, tolerable, durable therapies for metastatic melanoma.

## Data Availability

No datasets were generated or analysed during the current study.
